# Coral Skeletons Provide Historical Evidence of Phosphorus Runoff on the Great Barrier Reef

**DOI:** 10.1371/journal.pone.0075663

**Published:** 2013-09-27

**Authors:** Jennie Mallela, Stephen E. Lewis, Barry Croke

**Affiliations:** 1 Research School of Earth Sciences, The Australian National University, Canberra, Australian Capital Territory, Australia; 2 Research School of Biology, Australian National University, Canberra, Australian Capital Territory, Australia; 3 Catchment to Reef Research Group, TropWATER, James Cook University, Townsville, Queensland, Australia; 4 Fenner School of Environment and Society, Australian National University, Canberra, Australian Capital Territory, Australia; 5 Department of Mathematics, Australian National University, Canberra, Australian Capital Territory, Australia; Leibniz Center for Tropical Marine Ecology, Germany

## Abstract

Recently, the inshore reefs of the Great Barrier Reef have declined rapidly because of deteriorating water quality. Increased catchment runoff is one potential culprit. The impacts of land-use on coral growth and reef health however are largely circumstantial due to limited long-term data on water quality and reef health. Here we use a 60 year coral core record to show that phosphorus contained in the skeletons (P/Ca) of long-lived, near-shore *Porites* corals on the Great Barrier Reef correlates with annual records of fertiliser application and particulate phosphorus loads in the adjacent catchment. Skeletal P/Ca also correlates with Ba/Ca, a proxy for fluvial sediment loading, again linking near-shore phosphorus records with river runoff. Coral core records suggest that phosphorus levels increased 8 fold between 1949 and 2008 with the greatest levels coinciding with periods of high fertiliser-phosphorus use. Periods of high P/Ca correspond with intense agricultural activity and increased fertiliser application in the river catchment following agricultural expansion and replanting after cyclone damage. Our results demonstrate how coral P/Ca records can be used to assess terrestrial nutrient loading of vulnerable near-shore reefs.

## Introduction

Coral reefs and other near-shore ecosystems are under increasing pressure from land based sources of pollution (e.g. nutrient and sediment runoff), resulting in unhealthy ecosystems which are highly vulnerable to natural and anthropogenic disturbances [1-3]. Now, largely because of the combined influence of pollution, climate change and overfishing, many of the world’s reefs have lost their capacity to recover from natural disturbances such as storms or disease. As a result many reef ecosystems have undergone long-term phase shifts whereby corals die and fleshy macroalgae replace them [4,5].

Elevated levels of terrestrial runoff into the marine environment are among the most dire threats to coral reefs [1]. Human activities have altered river catchments, freshwater use, and the global phosphorus and nitrogen cycle [3] and the Great Barrier Reef (GBR) is no exception. European settlers to northern Australia began clearing forested areas for grazing and cropping in the 1860’s; subsequent agricultural land-use and fertiliser applications have caused increased soil erosion and nutrient runoff into the GBR [6,7]. Terrestrial runoff and nutrient enrichment (phosphorus and nitrogen) on coral reefs causes deteriorating water quality with subsequent reductions in coral growth and in extreme cases the demise of the entire reef [1,2,6,8,9].

Worldwide, the phosphorus loads of rivers have doubled largely due to agriculture and secondary activities such as deforestation, soil erosion and sewage runoff [6,10]. The near-shore zones of the GBR are regularly exposed to terrestrial phosphorus pollution which is thought to have a residence time of years to decades [11]. As a result pollutants exported to the GBR are likely to have had long-term impacts on reef development. Phosphate contamination negatively affects reef building corals by compromising reproduction, skeletal calcification and framework development [12-14]. To date, the paucity of long-term data makes it difficult to demonstrate a relationship between phosphorus exported by terrestrial runoff, phosphorus levels in the marine environment, and subsequent coral growth and reef development on the GBR. The aim of this study was to determine if phosphorus records in long-lived coral skeletons (P/Ca) were associated with adjacent catchment and riverine phosphorus records. Using a novel geochemical approach, we show that phosphorus levels in our coral skeletons have increased in recent decades on the central, in-shore GBR. We further demonstrate a strong positive relationship between P/Ca and 1) fertilizer-phosphorus applications and 2) riverine particulate phosphorus runoff.

## Materials and Methods

### (a) Study area

**Figure 1:**
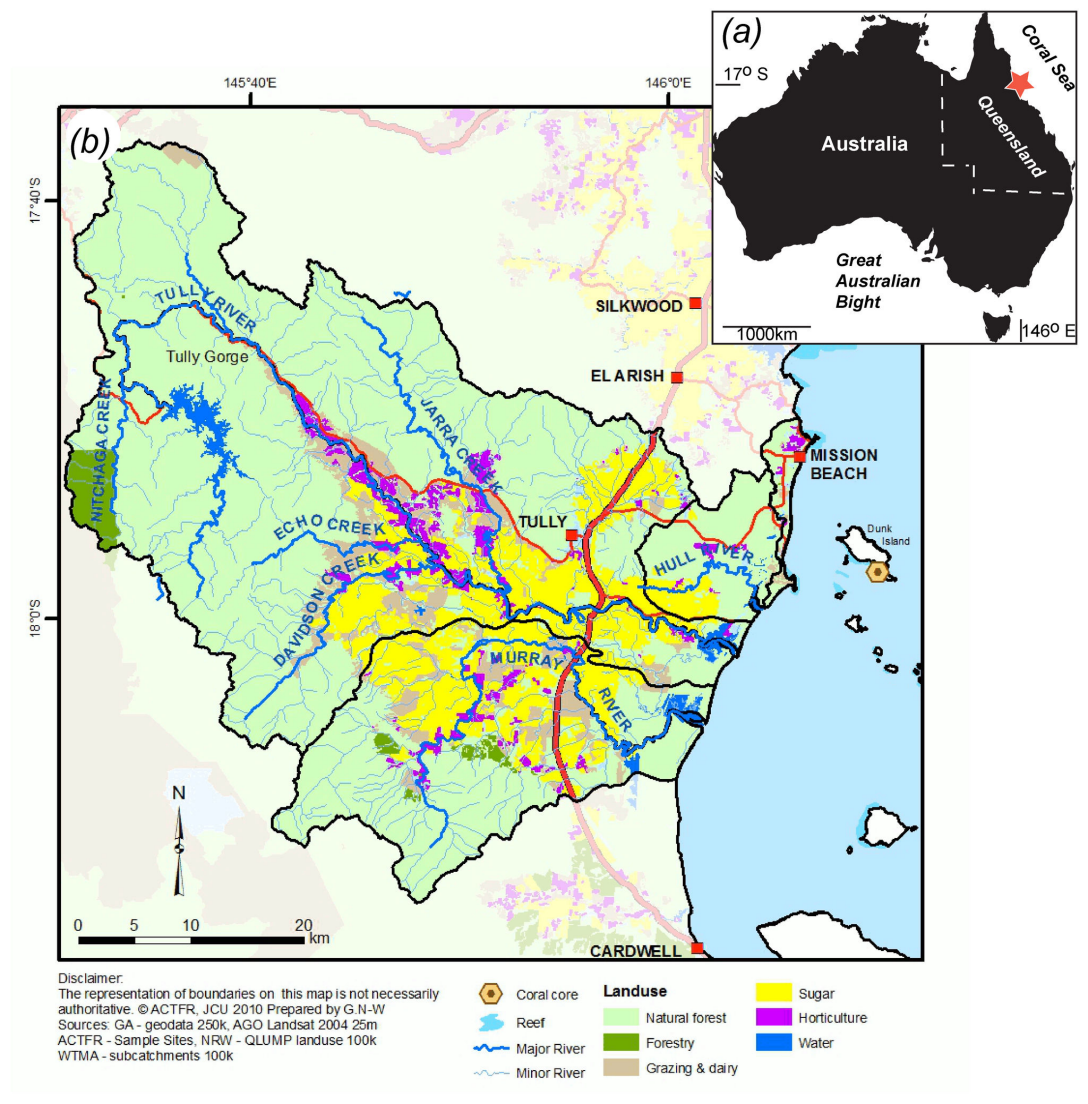
Map detailing (a) the location of the study area (red star) on the east coast of Australia and (b) land-use in the Tully River catchment and the location of Dunk Island coral cores.

Dunk Island (17°55 S, 146°10 E) is a continental island and nearshore reef in the central GBR (Figure 1a,b). Located 5 km from the mainland and 13.5 km to the north-east of the Tully River mouth, it is heavily influenced by terrestrial runoff, in particular sediment and nutrients [15]. Suspended sediment concentrations around Dunk Island have been reported to exceed 300 mg/L during turbid water events [16], with short-term sedimentation rates reported to be ~300g m^-2^ d^-1^ [17]. Dunk Island is composed of granite rock and surrounded by shallow fringing reefs with a well developed reef flat to the south. These turbid water reefs are bathymetrically restricted to shallow depths (

< 10m), despite this, the coral community is diverse [18]. However, recent cyclones and subsequent bleaching, disease and sediment smothering have resulted in hard coral mortality [19]. The island is regularly inundated by river runoff from the Tully River which brings pulses of turbid nutrient-rich waters throughout the year [15]. As a result reef sediments are characterized by a large proportion of siliclastic sediment [20].

**Figure 2:**
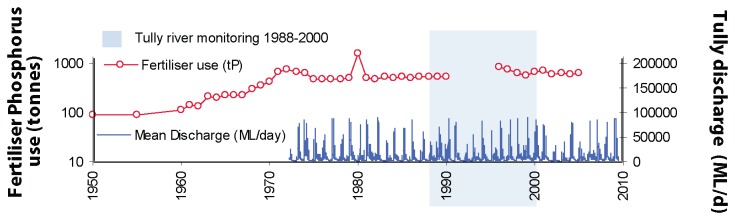
Time series detailing historical fertiliser-P use (tonnes, log scale) in the Tully River catchment 1950-2005 [21,22] and Tully River discharge 1975-2009 (data available: www.derm.qld.gov.au. Accessed 2013, Aug 26). Blue shaded boxes highlight the discrete 13 year period of contemporaneous river monitoring data.

The Tully River catchment lies to the south-west of Dunk Island and is located in the Wet Tropics. It drains an area of rainforest, intensive sugar cane and banana plantations (Figure 1b). Fertiliser-phosphorus applications across the Tully River catchment have increased 61 fold, from 10 tonnes in 1925 to 615 tonnes in 2005 [21,22], (Figure 2). Moreover, between 40-60% of freshwater wetlands in the Tully and adjacent Murray River catchments, which naturally trap sediment and nutrients, have also been drained for agricultural land use and urban development [6]. The Tully receives high rainfall throughout the year, averaging 4100 ± 1000 mm y^-1^ (± 1 SD) per annum from 1925 to 2009 [23]. River flow is continuous throughout the wet and dry season with frequent flood events and a mean annual flow of 3.5x10^6^ ML [24], (Figure 2).

Estimates of Tully River loads suggest dramatic increases since pre-European settlement with total suspended sediment increasing from 24 to 92 ktonnes y^-1^, and particulate phosphorus from 25 to 67 tonnes y^-1^ [7]. Sediment laden, nutrient-rich plumes from the Tully River typically move in a northerly direction such that Dunk Island corals are subject to terrestrial inputs from the Tully River catchment throughout the year, including between one and four flood water events annually [6,15]. The particulate phosphorus load originates largely from fertiliser-phosphorus use whereby phosphorus added to the soil binds with sediment and is exported from the catchment as a result of soil erosion and catchment runoff [6,25].

### (b) Historical data used in analyses

Annual records of fertiliser-phosphorus application were available from 1925 to 2005 for the Tully River catchment [21,22], with a small gap in the continuous record from 1991-1995 (Figure 2). Additionally, annual particulate phosphorus loads from the Tully River were obtained from a discreet 13 year monitoring program which occurred from 1988 to 2000 [25].

### (c) Coral collection

Permission to collect coral cores was obtained from the Great Barrier Reef Marine Park Authority. Three coral cores were collected from the southern end of Dunk Island in 2009 (Figure 1b) from three separate coral colony heads. Each core was collected from a healthy massive *Porites* colony with colonies located within 100 m of each other. Cores were collected at a water depth of 5m using a handheld pneumatic drill. Core barrels were 50cm long and 5.5cm in diameter. In the laboratory, cores were cut length-wise and sectioned into approximately 7mm thick slices, rinsed in freshwater and air dried (see File S1 for further details).

### (d) Coral chronology

**Figure 3:**
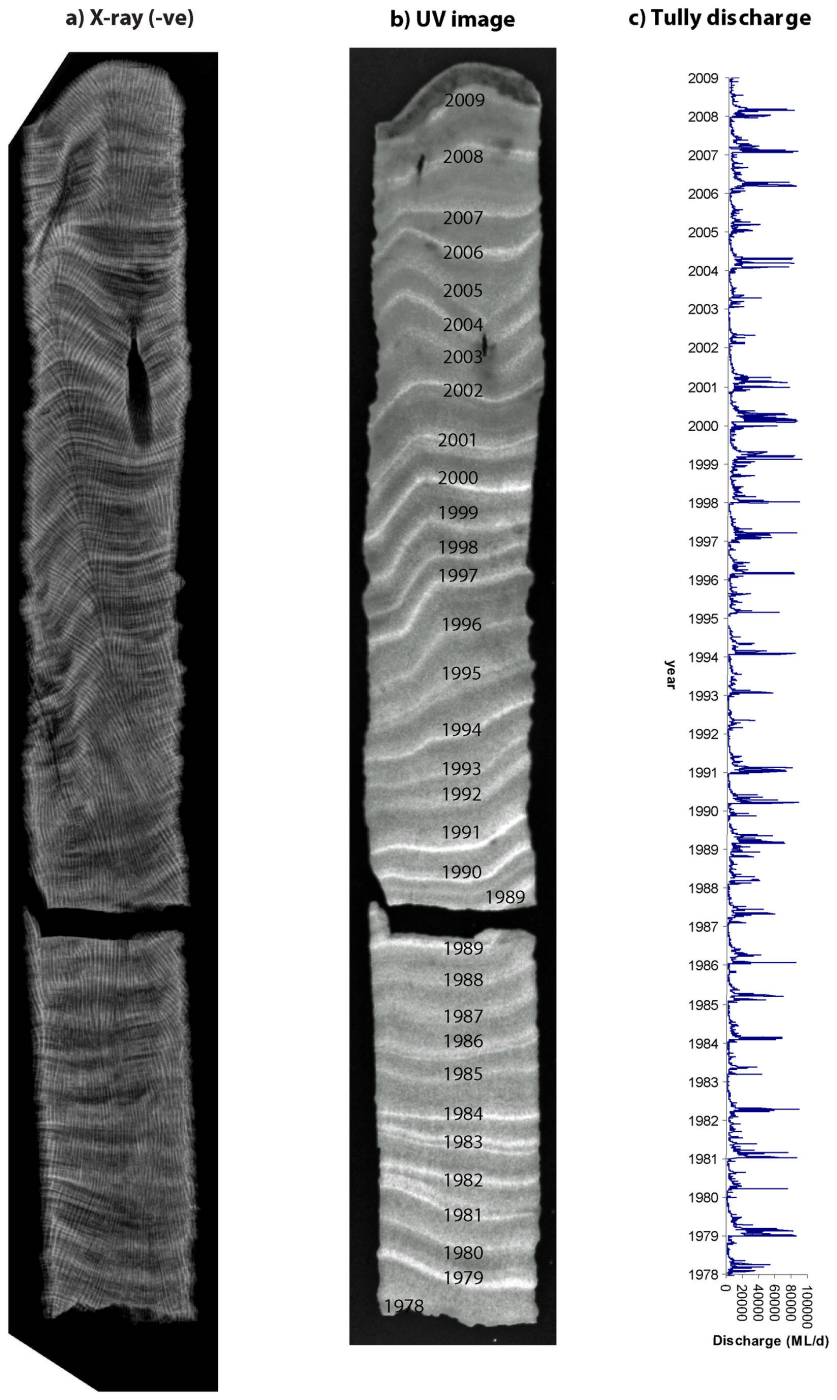
Coral chronologies determined from: (a) X-ray image displaying density bands; (b) image taken under UV light displaying luminescent lines of flood events, and (c) Tully river discharge (ML d^-1^).

The chronology of coral cores was assigned and cross checked by multiple dating techniques: 1) x-ray images of annual high density and low density skeletal bands were obtained from sectioned coral cores (Figure 3a), and the number of couplets counted [26]; 2) coral cores were examined under a UV light to reveal luminescent lines which correspond with freshwater flood events (Figure 3b-c) [27]; and 3) seasonal temperature geochemical proxies (Sr/Ca, U/Ca) were used to confirm summer and winter peaks and troughs. Luminescent lines, and their assigned year, in the coral archive were cross checked with Tully River discharge records (data source: www.derm.qld.gov.au) in order to verify assigned chronologies (Figure 3a-c). Additionally, seasonal temperature proxies were aligned with long-term regional seawater temperature records (data source: http://iridl.ldeo.columbia.edu). Coral core and external, historical data-sets were then imported into AnalySeries [28] for data matching and verification of assigned chronologies (see File S1 for further details).

### (e) Environmental records in coral cores: sediment and phosphorus

Geochemical signatures from the three coral cores were used to recreate phosphorus (P/Ca) and sediment (Ba/Ca) runoff records. Barium (Ba/Ca ratios) in coral skeletons from the GBR are known to document historical records of fluvial sediment export onto the reef [29,30]. Prior research [31-38] also indicates that phosphorus (P/Ca) records are also captured downcore in the calcium carbonate skeleton of tropical, hermatypic corals. We used laser ablation inductively coupled plasma mass spectrometry (LA-ICP-MS) to document in-situ skeletal P/Ca and Ba/Ca ratios in coral cores. The LA-ICP-MS technique is specifically adapted for coral cores [29,39]. It provides high resolution analyses of entire coral cores for reconstruction of trace element records at a monthly or annual resolution (see File S1). Phosphorus and barium were measured along clean, sliced sections of *Porites* using a Helex LA-ICP-MS system and the system specific methods detailed in [38,40]. In brief, pre-cut samples were thoroughly cleaned ultrasonically and then subjected to an initial laser ablation scan to additionally clean the sectioned coral surface, and to condition and stabilise the ICP-MS prior to analyses. Each repeated laser cleaning scan removed ~1 µm from the surface of the sample over a 500 µm wide band along a pre-defined analysis track. The coral samples were subsequently analysed using a rectangular laser slit 400 µm perpendicular to the growth axis and 40 µm wide, parallel to the growth axis, using the following settings: scan speed of 40 µm/s, 5 Hz pulse rate and ~5 J/cm^2^. Coral samples were bracketed using the glass standard NIST 614 (National Institute of Standards and Technology) and the in-house pressed powder coral standard. Raw data were smoothed using a 10-point running mean (see File S1 for further details).

Data obtained from the live tissue layer, within ~1cm of the growing surface of the coral core, were discarded [38] and only data obtained from tissue-free skeleton were used. Samples downcore were checked visually and electronprobe microanalyses was used to ensure remnant coral tissue had been fully removed [38]. Results are presented here for the period from 1949 to 2008 for one long core and are supplemented by results from two shorter coral cores which date back to the 1970’s.

### (f) land-sea relationships

Our aim was to see if there were any relationships between terrestrial phosphorus inputs and phosphorus on the reef (P/Ca). We therefore tested to see if there was a relationship between mean annual *Porites* phosphorus (P/Ca) and: 1) annual fertiliser phosphorus records; 2) annual Tully River particulate phosphorus records; and 3) annual fluvial sediment inputs (Ba/Ca).

Statistical analyses were conducted using SPSS 19. Normality of distribution and homogeneity of variance were tested through Kolmogorov-Smirnov and Levene’s tests, respectively. As data were not normally distributed Spearman rank-order correlation (r_s_) was used to test for relationships between P/Ca records and land runoff variables (fertiliser-P, Ba/Ca, river particulate phosphorus).

## Results and Discussion

**Figure 4:**
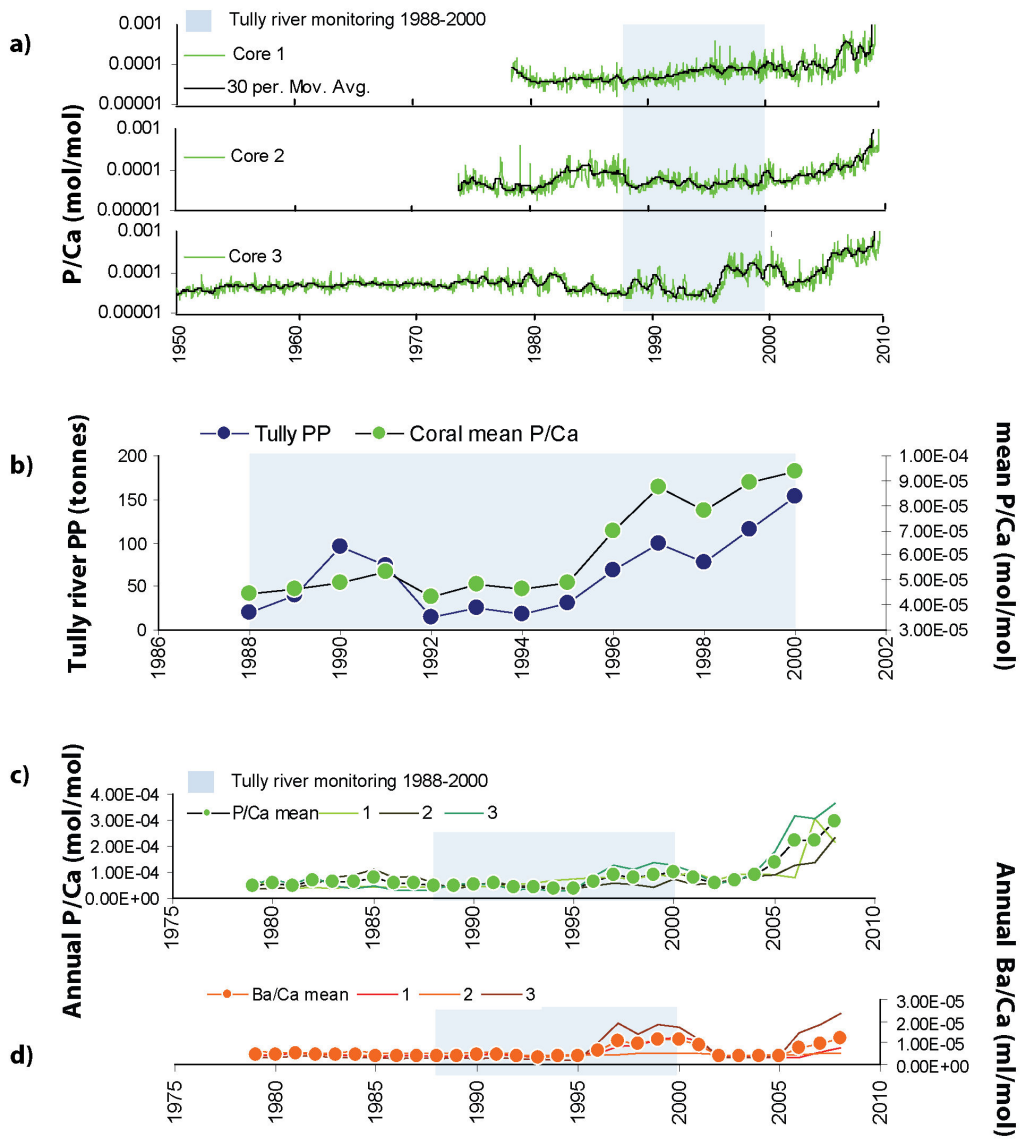
Time series detailing (a) fine resolution P/Ca (log scale, mol mol^-1^) records from 3 individual Dunk Island coral cores, (b) mean annual records for: P/Ca coral cores (n=3) and Tully River particulate phosphorus loads. Annual trace element records from each core (numbered 1 to 3) and annual mean values (n=3 cores) for (c) P/Ca (phosphorus proxy), and d) Ba/Ca (sediment proxy). Note: data from the live coral tissue zone have not been included. Blue boxes highlight the discrete 13 year period of contemporaneous river monitoring data.

**Figure 5:**
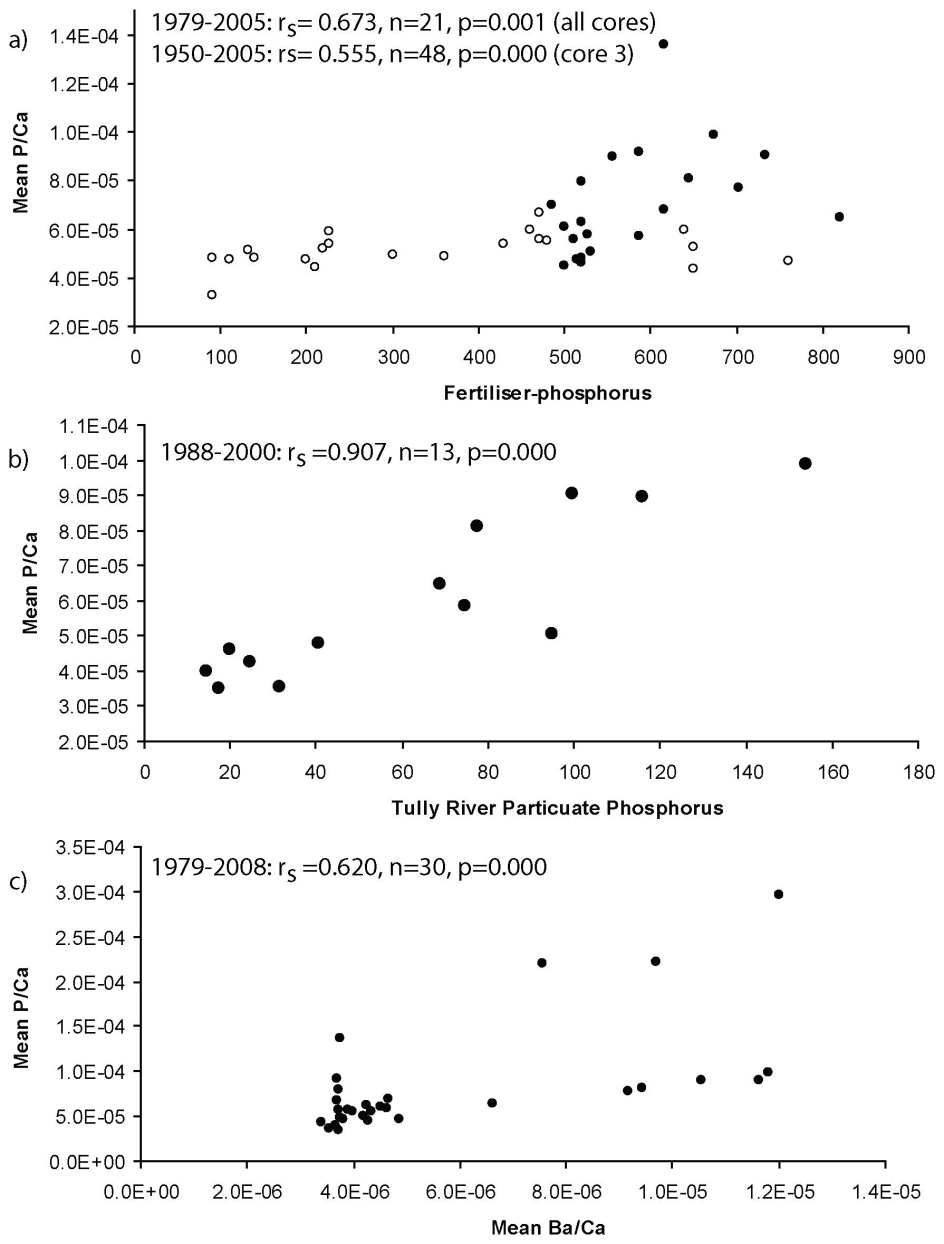
Scatter plots detailing mean P/Ca (mol mol^-1^) from *Porites* cores from Dunk Island and (a) fertiliser-phosphorus (tonnes), 1950-2005, open circles represent annual means from one long core 1950-1978 and filled circles represent annual means from all three cores from 1979-2005. Correlation detailed for the three core dataset time period (cores 1-3, 1979-2005) and separately for the longer time-frame (core 3, 1950-1978); (b) Tully River particulate phosphorus loads (tonnes) over 13 years, 1988-2000; and (c) Ba/Ca (mol mol^-1^) from 1979-2008. All data points represent annual means. Note all 3 graphs show highly significant correlations (p<0.01).

Data obtained from our three coral cores indicate that P/Ca and Ba/Ca ratios in corals from Dunk Island have increased over recent decades (Figure 4a-d). Mean annual P/Ca was also found to have a clear positive relationship with both annual fertiliser-phosphorus (Figure 5a) and riverine particulate phosphorus (Figure 5b). P/Ca ratios prior to the 1960’s display a consistent low level of phosphorus in the long coral core (≤ 0.11 millimol mol^-1^), (Figure 4a). In the early half of the 20^th^ century fertiliser-phosphorus applications were relatively low (year 1925: 10 tP) and increased steadily from the 1920’s (1930: 25 tP; 1940: 60 tP and 1950: 90 tP) [21] (Figure 2). From the 1960’s fertiliser-phosphorus application increased with a period of subsidised superphosphate sales and intensified sugar cane production, which rose from 110 tP in 1960 to 615 tP in 2005 [21,22] (Figure 2). Agricultural practices at the time included burning of sugar cane prior to harvesting resulting in increased amounts of topsoil erosion and nutrient loading [41]. Disturbance and clearance of aquatic vegetation within the Tully River floodplain also resulted in more soil erosion by flood waters and during storm events [6]. This is reflected in the annual phosphorus (P/Ca) and sediment (Ba/Ca) signals in the cores where elevated pulses of P/Ca correspond closely with a period of increased fertiliser-phosphorus use. Data obtained from the three cores spans the time period 1979-2005 with mean annual P/Ca positively correlated with fertiliser-phosphorous (1975-2009: Spearman’s rank: r_s_= 0.673, n=21, p=0.001, Figure 5a). Similarly, the longer P/Ca record obtained from the single long core (Figure 4a, core 3) also correlates with contemporaneous fertiliser records (1950-2005: Spearman’s rank: r_s_= 0.555, n=48, p=0.000, Figure 5a). Sediment runoff signals (Ba/Ca) also correspond closely with phosphorus records in cores (Figure 4c,d). The mean annual P/Ca was positively correlated with Ba/Ca for the last three decades (Spearman’s rank: r_s_ =0.620, n=30, p=0.000, Figure 5c) suggesting a particularly strong relationship between annual phosphorus and fluvial sediment loading in the marine waters surrounding Dunk Island.

At the end of the 1980’s much of the remaining natural wet tropical grasslands in the Tully River catchment were converted to intensive agricultural systems for harvesting sugar cane and bananas. During this time (1988 to 2000) the Tully River water quality was also routinely monitored for phosphorus [25] enabling comparison with P/Ca levels in the coral cores. Mean annual P/Ca ratios in coral cores are found to be strongly correlated with annual particulate phosphorus loads exported from the Tully River during this 13 year monitoring programme (Spearman’s rank: r_s_ =0.907, n=13, p=0.000) (Figures 4b and 5b). Large P/Ca increases occur in coral cores throughout the 1980’s and 1990’s and correspond with increased particulate phosphorus levels in the Tully River and with increased catchment fertiliser-phosphorus use [25].

The findings presented here support a growing body of work aimed at exploring the phosphorus signature captured in tropical corals and documenting anthropogenic nutrient enrichment. Some of the earliest studies of scleractinian phosphorus signatures come from the Caribbean: St Croix; Curacao; and Bermuda, where studies of *Montastrea* *annularis* and *Diploria* *strigosa* were undertaken [31]. The total phosphorus and inorganic phosphorus content of the skeleton was found in certain colonies to be elevated at dredged, sewage impacted, and phosphate ore loading sites. Whilst differences were observed between geographic locations and species they concluded that annual variation was primarily due to sewage impacts. Findings from Bermuda also suggested that P/Ca was similar to seawater ratios [31]. Subsequent work in Tobago [32] on *Montastrea* *annularis* also concluded that over 3 decades, historic records of phosphorus (total, inorganic and organic) in coral cores were linked to runoff. This was attributed to changing landuse in adjacent catchments (e.g. agriculture and development), terrestrial and sewage runoff. Research from Mauritius in the Indian Ocean [33] on *Porites* sp. also concluded that anthropogenic inputs, primarily sewage and livestock runoff, were responsible for elevated phosphate concentrations.

Recent short-term studies have also explored the coral P/Ca record in relation to seawater concentrations. A 4 year skeletal P/Ca record from a single *Pavona gigantea* coral colony was shown to vary with surface water phosphate concentrations [35] and a 13 month long experiment at an upwelling location in the Gulf of Panama found strong correlations between surface water PO_4_ and P/Ca in multiple coral colonies [36]. Studies of a single *Porites* colony from a eutrophic region of the China Sea also found that the P/Ca signal was strongly driven by seawater total phosphorus [37]; they concluded that their signal was derived from phosphate and organic phosphorus. The method of phosphorus incorporation, and the exact location of the phosphorus in the skeleton is still under investigation and warrants further attention. It seems likely that down-core, skeletal phosphorous in tropical corals may be present in various chemical forms. We hypothesise that in the absence of sediment being present in the coral skeleton, particulate phosphorus may be desorbed from the sediment to phosphate where it could be incorporated into the skeleton. This could happen via two mechanisms: 1. relatively fast desorption from the sediment within the flood plume itself, or 2. desorption at a latter period where the bottom sediments become oxygenated. The findings presented here have also been interpreted in light of earlier fine scale mapping of phosphorus in these samples [38]. High spatial resolution mapping of phosphorus in these *Porites* coral skeletons from Dunk Island on the GBR [38] reveal that phosphorus is present at much lower levels (<500 ppm) in the calcium carbonate coral skeleton when compared to the living tissue zone (up to 8700 ppm) requiring that skeletal compositions be only compared below the live tissue zone. Moreover, small local heterogeneities (

< 250 µm) occur in skeletal P/Ca that have the potential to bias fine resolution (e.g. weekly) records and make P/Ca time series data best interpreted at coarser (e.g. seasonal or annual) temporal resolution [38]. Whilst the organic portion of the skeletal matrix can contain phosphorus a large proportion of skeletal phosphorus (> 60%) in tropical corals has been found in the intra-crystalline, organic phases of the skeleton [35]. It has also been suggested that fine scale phosphorus heterogeneities may be due to micro-endoliths which are not removed during standard cleaning techniques [38]. It is interesting to note that intra-skeletal, micro-endoliths are more abundant in coral skeletons at river/nutrient impacted locations [42]. Clearly, the location of phosphorus in the coral and the role of biological and environmental controls warrants further attention in order to improve this promising phosphorus proxy at a finer level of resolution (e.g. sub-annual).

Here we demonstrate for the first time that mean P/Ca records from near-shore *Porites* skeletons are robust indicators of fluvial phosphorus runoff at the annual level. Phosphorus runoff is a major eutrophication threat to the Great Barrier Reef. In the absence of long-term spatio-temporal data, it has been difficult to document and assess pollution impacts. This hinders management of agricultural activity, fertiliser use and phosphorus runoff on coral reefs [43]. Annual P/Ca variations in coral cores may provide a proxy for long-term terrestrial phosphorus loading. We have demonstrated that *Porites* coral cores from the central GBR contain long-term records of phosphorus export from an adjacent catchment. Our findings indicate that annual mean phosphorus (P/Ca) records captured in coral cores from Dunk Island correlate closely with annual fertiliser-phosphorus use, fluvial sediment proxies (Ba/Ca) and riverine particulate phosphorus (PP) loads recorded in the Tully River catchment. We conclude that P/Ca levels in near-shore coral cores provide a useful long-term indicator of phosphorus export from adjacent catchments and particulate phosphorus loading into the near-shore GBR. This approach, if extended further back in time, could also provide valuable insights into how changing human-landscape interactions over the last two centuries have modified catchment to reef nutrient cycling. Current predictions indicate substantial increases are likely to occur in the frequency of intense storms and heavy rainfall events, with coastal regions becoming increasingly vulnerable to storm-surge flooding [44]. As a result extreme terrestrial runoff events containing elevated sediment and nutrients are likely to increase in the near future. The implications of this for catchment management, water quality and the continued health of the Great Barrier Reef are poorly understood and warrant immediate attention.

## Supporting Information

File S1 **A detailed methodology.** This file also includes Figure S1, Replicate LA-ICP-MS runs (n=4) to measure P/Ca.
(DOC)
