# Supplementary material for: Coral Skeletons Provide Historical Evidence of Phosphorus Runoff on the Great Barrier Reef
Source: PLoS One. 2013 Sep 27;8(9):e75663. doi: 10.1371/journal.pone.0075663 (PMC3785503; doi:10.1371/journal.pone.0075663)
Supplement: File S1 — A detailed methodology. This file also includes Figure S1, Replicate LA-ICP-MS runs (n=4) to measure P/Ca. (DOC) [file pone.0075663.s001.doc]

**S1. Supporting on-line material:**

**Detailed Methodology**

*Sample collection and treatment*

Three *Porites* coral colonies were cored in July 2009 at the south end of Dunk Island. Healthy colonies were selected for sampling according to: 1) depth (ca. 5 m), 2) morphology (uniform surface of colony), and 3) minimal evidence of internal bio-erosion, bleaching or disease. The water depth at the top of the colonies ranged from 3 to 4 m, and at the base of the colonies the depth was 4 to 5 m. Corals were cored using a hand held hydraulic drill, in sections approximately 50 cm long and 5.5 cm in diameter.

Immediately following collection, coral cores were washed in fresh water to remove salts. The upper live tissue layer of the coral cores was then submerged for 12-24 hours in dilute hydrogen peroxide (3%) to remove residual coral tissue and organic matter from the skeleton. Cores were then allowed to air dry in the sun. Prior to cutting in the laboratory the tissue zone was soaked again in dilute hydrogen peroxide (3 %) for 24 hours to remove any residual organic matter. Cores were cut lengthwise, along the growth axis into 7 mm thick slabs using a water lubricated rock saw. Slabs were rinsed in freshwater and air dried. The least taphonomically altered sections from each colony were selected for analyses. Any sections displaying high rates of bioerosion or any growth abnormalities were discarded.

*Density bands and flood bands*

X-ray radiographs were taken using standard hospital X-ray equipment to reveal the annual density bands in all coral core sections. The film was X-rayed at 50 KvP and 10 mA with a source to object distance of 1.25 m for 5 to 15 seconds based on slab thickness and density. Luminescent lines were photographed under an ultra violet (UV) light (principal wave length 365 nm determined at 200 mm from source surface) using an Olympus digital camera.

X-ray images and UV images were examined visually and those displaying clear banding patterns were selected for analysis by laser ablation inductively coupled plasma mass spectrometry (LA-ICP-MS). Coral slices were then cut into contiguous pieces with a maximum length of 9.7 cm and width of 2.5 cm along the plane of maximum growth using a bandsaw in order to fit into the laser ablation chamber.

*LA-ICP-MS*

Analytical methods were adapted from prior LA-ICP-MS coral core research . As samples were being analysed for phosphorus (P/Ca) additional steps were taken to ensure samples were thoroughly cleaned and ablated. This included: 1) an additional ultrasonic bath cleaning step (based on results of a pilot study, see Figure S1 in file S1); and 2) an additional laser ablation cleaning run was introduced.

1. ***Ultrasonic bath***: Coral pieces were first cleaned individually in an ultrasonic bath (Techspan L33oH) with Milli-Q water (18 mega ohm). They were cleaned for a total of 30 minutes with water being exchanged 3 times (3 x 10 minute cleaning periods per sample).
2. ***Ultrasonic probe***. Samples were then cleaned using an ultrasonic probe which allowed us to focus the ultrasonic beam along the pre-designated analyses track (Homogeniser 4710 series, % duty cycle: 70). The pieces were cleaned in Milli-Q water using an ultrasonic probe to remove any coral powder or debris left from the rock sawing process. The output level of the ultrasonic probe was left below 2 to avoid the destruction of the microstructures of the coral skeleton. Pieces were then left to dry overnight in an oven set at a 40°C.

*LA-ICP-MS analyses*

An ANU HelEx laser ablation system (based on a Compex 110i ArF laser) linked to a quadropole ICP-MS (Varian 820 MS) was used to measure B/Ca, P/Ca, Sr/Ca, Ba/Ca, U/Ca in selected slices of coral core sections .

*Laser ablation cleaning*

***Cleaning run 1***: Prior to analyses the samples were cleaned by placing coral pieces in the sample stage with a standard pre-ablating sequence: slit size 500 x 40 µm, a 10 Hz pulse rate and scanning at 100 µm/s.

***Cleaning run 2:*** Pilot studies indicated that for phosphorus (P/Ca) an additional ablation cleaning run is required, settings used: rectangular slit 400 µm perpendicular to the growth axis and 40 µm wide parallel to the growth axis, scan speed: 40 µm/s, 5 Hz pulse rate and 50MJ energy. After sampling tracks were ablated a second time samples were analysed using LA-ICP-MS coral settings detailed in the section below.

***Analyses run:*** coral pieces are placed in a sample stage, and scanned at 40 µm/s using 5 Hz pulse rate and 50MJ energy. A rectangular slit 400 µm (perpendicular to the growth axis) and 40 µm wide (parallel to the growth axis) is used to mask the laser beam. Coral sample analyses are bracketed between analyses of a glass standard (NIST614) and an in-house coral standard from a coral from Davies Reef . Raw data were smoothed using a 10-point running mean. The mean background (±1SD) for 31P and 43Ca was 6435 (±435) and 7 (±14) 14 counts s^-1^, respectively. Conservative detection limits based on the mean (±6SD) of the background count rate were 7539 (±0.96) for ^31^P and 213296 (±64.4) ppm for ^43^Ca with a peak to background ratio on the coral standard of ^31^P\20, and ^43^Ca>10,000 (as detailed in ).

***Pilot study to test data reproducibility:*** As P/Ca analyses using LA-ICP-MS is a relatively new technique pilot studies were conducted to check data were reproducible using the above detailed cleaning and pre-ablation method. During the pilot study random laser ablation tracks were selected and then identical laser tracks were repeatedly set and rerun up to four times. Data were then compared between the four identical LA-ICP-MS runs. It was concluded that whilst trends between runs were similar run 1 showed slightly elevated levels of P/Ca. As the data from runs 2, 3 and 4 were not significantly different (Figure S1 in file S1), we concluded that run 2 should be the standard approach. As a result of these findings our analyses method incorporated an additional laser cleaning run (cleaning run 2) prior to data collection. LA-ICP-MS data were also crosschecked with electronprobe microanalyses (EMPA) data in an early pilot study .

Figure S1 in file S1. Replicate LA-ICP-MS runs (n=4) to measure P/Ca

*Sclerochronology*

#### ***Coral chronology and linear extension:*** Coral cores were aged using multiple dating techniques which included: 1) annual density bands obtained from X-rays; 2) luminescent lines of river discharge events; and 3) seasonal trace element signatures (Sr/Ca, U/Ca, B/Ca). Typically the results from the x-rays and luminescent lines were compared against Tully River discharge data (data available: www.derm.qld.gov.au. Accessed 2013, Aug 26) and seasonal geochemical temperature proxies obtained from the LA-ICP-MS compared with long-term regional sea water temperature records ([http://iridl.ldeo.columbia.edu](http://iridl.ldeo.columbia.edu/). Accessed 2013, Aug 26). Results were cross-checked manually and using the time series analyses program AnalySeries .

Annual linear extension (growth: mm y^-1^) was calculated using: 1) the assigned dates from cross dating, 2) measuring the distance between assigned years, and 3) all dates and analyses were based on the distance along the maximum growth axis. For the geochemical data the distance was calculated between the seasonal peaks or troughs of the annual cycles of Sr/Ca, Ba/Ca, U/Ca and/or B/Ca. The principle for the X-rays was similar whereby the distance between density bands was measured along the major growth axis. The time series analyses program AnalySeries was used to identify annual and seasonal markers (e.g. summer temperature maximum) using the three methods outlined above. Subsequently this data was used to calculate annual linear extension rates.

*Data analyses*

Records from the dead coral skeleton were used for all reported data analyses. Data from the top end of the coral in the live tissue zone were collected but not used in analyses as this combines trace element signatures in the tissue zone and gives anomalously high values . P/Ca data was resampled at regular time intervals (e.g. annual) using the computer programme AnalySeries in order to look for relationships between P/Ca and annual records for fertiliser-P and riverine particulate P.

Daily river discharge data were downloaded from [www.derm.qld.gov.au](http://www.derm.qld.gov.au/). Cross correlations with river discharge were conducted to assess time lags in P signals from catchment to reef. Cross correlations were performed using the daily stream flow data and weekly P/Ca ratios. The P/Ca data were converted to daily by infilling with negative values due to the variable temporal sampling of P/Ca data (ranging from 7 to 10 days). This occurs as corals do not grow at a constant rate, so whilst LA-ICP-MS was conducted at regularly spaced increments down-core, each increment does not necessarily equate to the same period of time. The negative values inserted into the  P/Ca data are treated as falgs, with the algorithm ignoring these in the calculation of the cross correlation functions. This treats the data points as distinct observations, but enables the analyst to look at the full resolution stream flow data. Due to the presence of missing data, where core sections were joined together, the Fourier transform approach for calculating the cross correlation function could not be used. Rather, the cross correlation function was determined using the approach described in . For each lag, all valid pairs of flow (not negative) and P/Ca ratios were combined into a pair of vectors, and normalised to have a mean of zero, and a standard deviation of 1. The sum of the product of the normalised pairs was then calculated (the autocorrelation of flow was also calculated using the vector of normalised selected flow data). This approach is computationally inefficient, but able to handle incomplete datasets. The underlying assumption is that the available data give a reasonable indication of the cross correlation function. The resulting cross correlation functions were then smoothed using a box-car filter of width 15 days.

An autocorrelation of flow shows the seasonal variation in the discharge. The cross correlation between the P/Ca ratios and the stream flow shows that there is a delay in the peak in the P/Ca ratio compared with the discharge that varies with location. Core 1 appears to have the shortest delay (earliest peak in the cross correlation function) and core 2 the longest delay. In all cases, the seasonality decays with time, suggesting that the P/Ca ratio is more event dependent, and less dependent on the total volume of wet season discharge. Results demonstrated a weak relationship, but clearly highlight a time delay of several months between river flow and down core P records.

Relationships between mean annual P/Ca records from the 3 coral cores and existing published catchment records for fertiliser-P applications and riverine particulate phosphorus (PP) were tested using: 1) Pearson product-moment correlation (r) for Tully River PP v mean P/Ca, and 2) Spearman rank-order correlation (r_s_) for fertiliser-P use v P/Ca. Data analyses met the standard assumptions.

References

1. Alibert C, Kinsley L (2008) A 170-year Sr/Ca and Ba/Ca coral record from the western Pacific warm pool: 2. A window into variability of the New Ireland Coastal Undercurrent. Journal of Geophysical Research 113: 1-C06006.

2. Alibert C, Kinsley L, Fallon SJ, McCulloch MT, Berkelmans R, et al. (2003) Source of trace element variability in Great Barrier Reef corals affected by the Burdekin flood plumes. Geochimica et Cosmochimica Acta 67: 231-246.

3. Fallon SJ, McCulloch MT, van Woesik R, Sinclair DJ (1999) Corals at their latitudinal limits: laser ablation trace element systematics in Porites from Shirigai Bay, Japan. Earth and Planetary Science Letters 172: 221-238.

4. Sinclair DJ (1999) High spatial-resolution analysis of trace elements in corals using laser ablation ICP-MS. PhD, Australian National University.

5. Mallela J, Hermann J, Rapp R, Eggins S (2011) Fine-scale phosphorus distribution in coral skeletons: combining X-ray mapping by electronprobe microanalysis and LA-ICP-MS. Coral Reefs 30: 813-818.

6. Paillard D, Labeyrie L, Yiou P (1996) Macintosh program performs time-series analysis. Eos Transactions 77: 379.

7. Croke. BFW. Land use impacts on hydrologic response in the Mae Chaem catchment, Northern Thailand, . V. Kachitvichyanukul, U. Purintrapiban, P. Utayopas, eds., Bangkok, Thailand, January 17-19; 2005. pp. 434-439.

8. Pulsford JS (1996) Historical nutrient usage in coastal Queensland river catchments adjacent to the Great Barrier Reef Marine Park. Research Publication 40.

9. Brodie J (2007) Nutrient management zones in the Great Barrier Reef catchment: a decision system for zone selection: report to the Department of Environment and Heritage. Australian Centre for Tropical Freshwater Research (ACTFR) report 06/07, James Cook University, Townsville, Australia.

10. Mitchell AW, Reghenzanl JR, Furnas MJ (2001) Nitrogen levels in the Tully River: a long-term view. Water Science and Technology 43: 99-105.
